# The Integration of Microwave-Synthesized Silver Colloidal Nanoparticles into Poly (Lactic Acid)-Based Textiles as Antimicrobial Agents via Pre- and Post-Electrospinning Processes

**DOI:** 10.3390/polym16243613

**Published:** 2024-12-23

**Authors:** Muhammad Omer Aijaz, Ibrahim A. Alnaser, Md Irfanul Haque Siddiqui, Mohammad Rezaul Karim

**Affiliations:** 1 Center of Excellence for Research in Engineering Materials (CEREM), Deanship of Scientific Research (DSR), King Saud University, Riyadh 11421, Saudi Arabia; 2 The King Salman Center for Disability Research, Riyadh 12512, Saudi Arabia; 3 Department of Mechanical Engineering, College of Engineering, King Saud University, Riyadh 11421, Saudi Arabia

**Keywords:** electrospinning, poly (lactic acid), silver nanoparticles, nanofibers, antibacterial, hydrophilicity, protective textiles, disability

## Abstract

This study introduces a novel method to enhance the antibacterial functionality of electrospun nanofibrous textiles by integrating silver nanoparticles (AgNPs) into poly (lactic acid) (PLA) fabrics through pre- and post-electrospinning techniques. AgNPs were incorporated into hydrophobic and modified hydrophilic PLA textiles via pre-solution blending and post-solution casting. A PEG-PPG-PEG tri-block copolymer was utilized to enhance hydrophilicity and water stability, while AgNPs served as antibacterial agents. Morphological analyses confirmed uniform, smooth, and beadless nanofibers with diameters between 435 and 823 nm. Energy-dispersive X-ray spectroscopy spectra and elemental analysis verified the successful incorporation of AgNPs, with higher Ag content in the post-electrospinning samples. Contact angle measurements showed an improved hydrophilicity of the modified PLA textiles, absorbing water droplets within 2 s. The X-ray crystallography patterns confirmed the amorphous structures of the PLA and PEG-PPG-PEG, with reduced crystallinity in the samples containing AgNPs. Thermal analysis indicated lower decomposition temperatures for the hydrophilic samples due to the plasticizing effects of PEG-PPG-PEG on PLA. Mechanical testing showed comparable tensile strengths but reduced elongation in the post-treated samples. The antibacterial efficacy was assessed against various bacterial strains, with post-electrospinning AgNP incorporation showing the most effective antibacterial properties. The results indicate that integrating electrospinning and nanofiber modification techniques expands the applications of PLA-based protective fabrics for disabled individuals.

## 1. Introduction

The scientific community studying membrane technology has recently become aware of the negative consequences of polymers made from petroleum on both human health and the environment. In 2019, the European Bioplastics and Nova Institute issued general guidelines urging the chemical industry to develop sustainable products by increasing the use of bio-based materials [1]. This awareness is expected to lead to a gradual shift from petroleum-based to bio-based polymers. Biodegradable polymers, such as cellulose acetate (CA), polyvinyl alcohol (PVA), polyvinyl acetate (PVAc), polycaprolactone (PCL), poly (lactic acid) (PLA), chitosan, and polyethylene glycol (PEG), have garnered significant interest because of their benefits over their petroleum-based competitors and biocompatibility [2,3,4].

With regard to biopolymers that can replace petroleum-based polymers, PLA is the most promising because of its high processability, cost-effectiveness, extensive availability, and biocompatibility. PLA is hydrophobic, has low heat stability, a moderate rate of degradation, low side-chain reactivity, and limited hardness. PLA is a known ecologically benign polymer in commercial applications, where it is used to produce biomedical goods, including joints, screws, scaffolds, and artificial blood arteries, as well as short-lived items, such as eating utensils, bottles, and packaging materials [4]. Additionally, it is used in agricultural applications, such as mulching films.

Extremely thin nanofiber membranes with unique properties that are appropriate for a range of applications, including tissue engineering, wound care, protective gear, wastewater treatment, and biomedical applications, have been produced over the past few decades via electrospinning techniques [5,6]. Fibers are produced by the electrostatic drawing of polymer jets during electrospinning. The process starts with the high-voltage stretching of polymer solution droplets, which produces nano- or microfibers that gather on the surface. The use of PLA-based electrospun fibers in scaffolds [7], drug delivery systems [8], tissue engineering [9], medical implants, protective textiles, wound dressings [10], water harvesting [11,12], and water treatment applications [13] has attracted a lot of interest. Therefore, PLA has become a very desirable and affordable nanofibrous material for usage in safety equipment due to its capacity to closely resemble the extracellular matrix and provide a sizable specific surface area, along with its high porosity, small pore size, and appropriate mechanical characteristics.

Attires fulfill multiple purposes, including safeguarding against weather, providing security, ensuring comfort, and expressing identity [14]. Appropriate clothing can boost an individual’s self-assurance in a social environment. Nevertheless, over a billion disabled individuals globally find it difficult to find clothing that satisfies their psychological and physical needs [15]. The standard of living for disabled and elderly individuals can often be enhanced by the use of aesthetically appealing and practical textiles. Garments for people with disabilities frequently require specific functional modification. The regulation of skin moisture transfer is a key consideration in this context. The degree of breathability of garments for use indoors and outdoors is determined by their water vapor transfer capability. Breathable attire allows excess heat to escape with moisture evaporation through tiny pores. Heat and moisture are trapped by non-breathable materials, such as plastic, dense pile fabrics, and thick, multi-layered textiles, resulting in discomfort, damp skin, and skin abrasion. This environment can encourage Gram-negative bacterial growth, increasing the likelihood of decubitus [16]. Individuals with sensitive skin, often exacerbated by conditions like muscle disorders and diabetes, require clothing with high tactile properties. The inclusion of antimicrobial additives in textiles can inhibit bacterial proliferation and prevent illnesses.

For both indoor and outdoor activities, breathability and water resistance are crucial. Nanofiber (NF) fabrics effectively wick moisture away from the skin, benefiting disabled individuals, babies, and older people, who might have difficulty with personal care duties because of trembling or poor muscle control. The selection of materials is vital, especially for protective clothes, such as nappies or bibs, to prevent staining from food and drink spills, which can irritate the skin and are challenging to remove. Membrane hydrophilicity is a crucial requirement for the effective use of nanofibrous membranes in fabrics. In the field of bio-medicated textiles that fight bacteria, water-loving surfaces promote interaction between cells and the membrane interface. Similarly, for optimal antibacterial performance, hydrophilic surfaces improve the membrane’s antimicrobial properties by permitting aqueous solutions to wet and subsequently infiltrate the fabrics. However, PLA, a hydrophobic polymer with a contact angle exceeding 120°, needs modification or functionalization to enhance its wettability (reducing the contact angle to below 90°) while maintaining water stability.

The development of hydrophilic characteristics in PLA-based nanofibrous textiles involves two main approaches: pre- and post-electrospinning. Prior to electrospinning, hydrophilic polymers, such as polyethylene glycol (PEG), chitosan, polyvinyl alcohol (PVA), and polyethylene oxide (PEO), are blended into the PLA mixture. In contrast, post-electrospinning approaches focus on the surface modification of electrospun PLA membranes using various wet chemical functionalization procedures. These techniques include coating, plasma treatment, grafting, hydrolysis, and multi-layered construction. The fundamental goal of these post-electrospinning approaches is to improve the hydrophilic characteristics of the nanofiber surface after electrospinning, thereby increasing the overall hydrophilicity of the PLA-based nanofibers [17,18,19,20].

Post-electrospinning techniques preserve the final characteristics of the scaffolds by just altering the fiber surface [21]. For example, the research conducted by Giulia et al. [22] employed both pre- and post-electrospinning techniques to enhance the antimicrobial activity in wound dressing applications. They first incorporated essential oils and then applied a chitosan coating to transform a hydrophobic PLA into a water-loving hydrophilic one. This hydrophilic surface not only amplified the antimicrobial efficacy of the essential oils but also promoted cell adhesion and growth.

In contrast, pre-electrospinning methods can alter hydrophilicity and improve additional features by adding other polymers, medications, and nanoparticles. Leila et al. [23] created curcumin-loaded PLA nanofibers for drug release in wound dressings. They achieved hydrophilicity by blending several percentages of PEG, which allowed faster drug release. Similarly, Nadia et al. [24] developed PLA-based nanofibrous scaffolds that incorporated PEG and multi-walled carbon nanotubes (MWCNTs) to transport anticancer medicines. PEG modified the PLA nanofiber properties, while MWCNT controlled the drug release. Another study focused on improving the hydrophilicity of PLA nanofibers using poly (lactic acid)-block-poly(ethylene glycol) (PLA-b-PEG) copolymers and biotin functionalization to enhance biosensor properties. The biotin availability on the PLA surface increased from 11% to 60% because of the PLA-b-PEG copolymers. This modification also improved the water stability of the PLA, making it suitable for short-term sensing applications in aqueous solutions [25]. In a separate study, low-molecular-weight Pluronic (a copolymer based on PEO) was combined with PLA to enhance its surface hydrophilicity. However, after 24 h in water, the membrane surface showed distortion and numerous holes due to PEO leaching from the fibers [26].

Most current research has mainly focused on PLA-based nanofibrous textiles, exploring various modifications and potential applications. A significant emphasis has been placed on improving the hydrophilicity and antibacterial properties of these materials, with the aim of developing advanced functional and protective clothing. This focus reflects the growing demand for high-performance textiles that can offer enhanced protection and comfort in various settings, from healthcare to industrial environments. To address the limitations of existing approaches and further advance the field, this research proposes an innovative strategy that evaluates both pre- and post-electrospinning techniques. This approach aims to synergistically enhance the functionality of electrospun nanofibrous textiles, particularly for antibacterial applications. This integration approach could lead to the development of more effective and versatile antibacterial textiles, opening up new possibilities for applications in medical textiles, protective gear, and other areas where bacterial resistance is crucial.

This study incorporated silver nanoparticles (AgNPs) into both hydrophobic poly (lactic acid) (PLA) and modified hydrophilic PLA fabrics using pre-and post-electrospinning methods via pre-solution blending and post-solution casting methods. To increase the hydrophilicity and water stability, a PEG-PPG-PEG tri-block copolymer was employed, while AgNPs served as antibacterial agents in the textile matrix. Various tests were conducted to confirm the changes in the morphology, surface wettability, chemical composition, and thermal stability of the PLA composite nanofibrous textiles. Additional tests ensured the structural and mechanical properties. The antibacterial efficacy of the PLA-based fabrics was also assessed. The results indicated that enhancing the hydrophilic properties of PLA nanofibers improved their water penetration efficiency, suggesting that the modified PLA could be a promising candidate for antibacterial applications. Combining electrospinning with nanofiber modification techniques may lead to a rise in the use of PLA-based textiles in drug delivery, scaffolding, disease management, medical devices, and pharmaceutical applications.

## 2. Experimental Methods

### 2.1. Material

Various materials were used in this study. Poly (lactic acid) (PLA, LX 175) was obtained from Filabot (Tokyo, Japan). Sigma-Aldrich supplied dichloromethane (DCM), dimethylformamide (DMF), and poly (ethylene glycol)-block-poly(propylene glycol)-block-poly(ethylene glycol) (PEG-PPG-PEG), with an average Mn of approximately 14,600. Additionally, silver nitrate and isopropanol (≥99.5%, ACS reagent grade, also known as isopropyl alcohol) were also provided by Sigma-Aldrich. Polyvinylpyrrolidone (PVP), with an average Mn of 44,000, was obtained from BDH. To create a laboratory-made silver colloid approximately 25 nm in size for incorporation into the PLA matrix, silver nitrate, isopropanol, and PVP were employed [27].

### 2.2. Preparation of Silver Colloids

Two separate solutions were prepared: 0.5 M AgNO_3_ in distilled water and 5 wt.% PVP in propanol. After ensuring that both solutions were homogeneous, they were mixed in equal parts in a reaction vessel. The sealed vessel was placed in a CEM MARS-4 microwave reactor. The reaction was carried out at 120 °C with a ramp time of 30 min and hold time of 15 s. The production of silver colloids and their antimicrobial efficacy were validated in our patented study, as referenced in [27].

### 2.3. Preparation of PLA-Based Hydrophobic and Hydrophilic Dope Solutions

Electrospun nanofibrous textiles were fabricated using a blended dope solution consisting of PLA and PEG-PPG-PEG. The first step in preparing a clear, transparent PLA solution was to dissolve a 12 weight percentage (wt. %) of PLA in DCM for 1 h at 50 °C. To prepare the PEG-PPG-PEG solutions, 10 wt.% PEG-PPG-PEG was added to DMF and heated to 50 °C for 30 min. Pure PLA and PEG-PPG-PEG solutions were prepared, combined at a 4:1 ratio, and stirred for 12 h to ensure that a homogenous solution was generated. Hydrophilic nanofibrous fabrics were prepared using a mixture of the PLA and PEG-PPG-PEG solutions, whereas hydrophobic fabrics were prepared using a pure PLA solution.

### 2.4. Integration of Silver Colloid into PLA-Based Textiles

#### 2.4.1. Pre-Electrospinning Stage

To enhance the antibacterial properties, a 20 *v*/*v* % silver colloidal solution was added to the pure PLA and blended PLA/PEG-PPG-PEG solutions. To ensure both homogeneity and dispersion, the AgNP-containing solutions were magnetically stirred for the entire day before the electrospinning procedure. Prolonged magnetic stirring ensured the uniform distribution of silver nanoparticles in the solution, preventing aggregation and settling. This homogeneity is crucial for producing consistent nanofibers with well-integrated AgNPs, maintaining dispersion stability, and preventing the nozzle from clogging during the electrospinning process.

#### 2.4.2. Electrospinning Process

The prepared pure PLA, PLA/PEG-PPG-PEG, PLA/AgNPs, and PLA/PEG-PPG-PEG/AgNPs dope solutions were added one at a time to a 10 mL syringe nozzle fitted with a 23-gauge needle for electrospinning. The machine (MECC, Fukuoka, Japan; model NF-500) was operated at a voltage of 21 kV until the 10 mL syringe was empty. During the electrospinning process, the needle was positioned at 15 cm from the flat collector, with a solution flow rate of 0.8 mL/h, while the ambient humidity was kept at 10%. Following the electrospinning, the nanofibrous fabric was detached from the collector and then dried overnight at 45 °C in an electrical oven. The samples were then preserved for characterization using various labels, as shown in Table 1.

#### 2.4.3. Post-Electrospinning Stage

**Figure 1:**
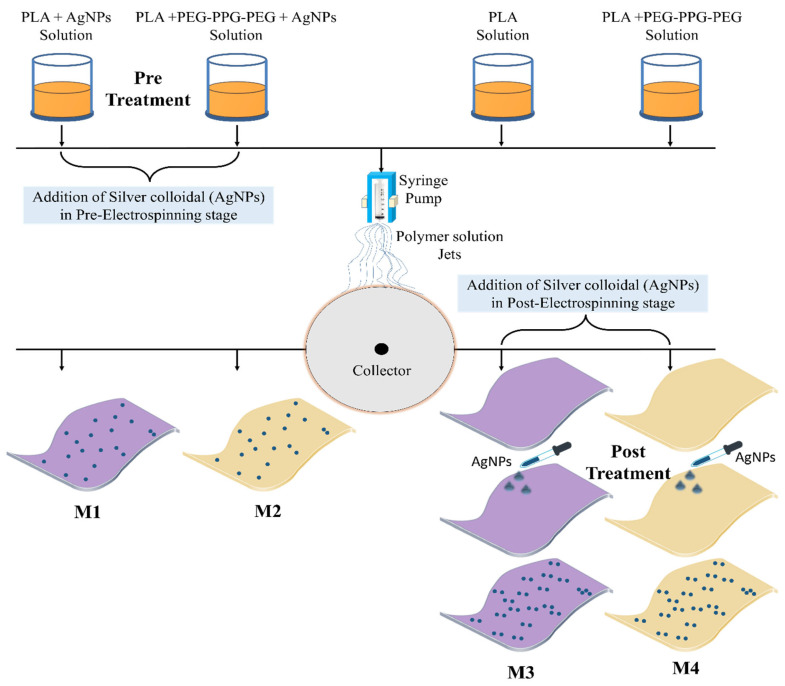
The preparation of the electrospinning dope solution and electrospun nanofibrous textiles, and the integration of silver colloidal into the textiles at the pre-and post-electrospinning stages.

The M3 and M4 membranes prepared using the pure PLA and PLA-PEG-PPG-PEG dope solutions were cut into small pieces and placed under a fixture with a diameter of 2 cm. A silver colloidal solution was added dropwise to the membrane surface, as shown in Figure 1. After 30 min, the membrane position was changed to avoid sticking to the surface, and the membrane was allowed to dry for 24 h at room temperature. The dried samples were stored for further characterization.

### 2.5. Characterization

#### 2.5.1. Morphological Study

Field-emission scanning electron microscopy (FE-SEM; JSM-7600, JEOL, Tokyo, Japan) was utilized to explore the surface morphologies of the fabricated M1, M2, M3, and M4 electrospun fabrics. Before the samples were placed inside the FE-SEM apparatus for morphological analysis, they were cut into small fragments and taped with carbon. A platinum-coated stub containing the specimen was used to boost the sample’s electrical conductivity during this study. The sputtering process was carried out at 30 mA for 60 s, resulting in a sputtering thickness of ~18 nm. The coated samples were then examined under a strong vacuum using FE-SEM.

The measurement of the NF dimensions involved two methods. First, the built-in software for the FE-SEM was utilized to measure the diameters of the 10–20 NFs. Second, Adobe Photoshop CS6 was used to determine the diameters of approximately 50 randomly chosen NFs. The collected measurements were then used to generate statistical graphs depicting the normal distribution patterns of the fiber dimensions.

#### 2.5.2. Crystalline Structure and Thermal Stability

The functional groups, amorphous or crystalline structures, and thermal behavior of the produced PLA-based fabrics were determined after the membranes were successfully fabricated. Utilizing a Fourier-transform infrared (FT-IR: VERTEX-70, Bruker, Billerica, MA, USA) apparatus, the functional groups were identified on a 600–4000 cm^−1^ scale. A thermogravimetric analysis apparatus (TGA Q600, TA Instruments, New Castle, DE, USA) was used to monitor the thermal behavior. A small part of the membrane was placed in a ceramic pan, and the test was conducted under an inert (nitrogen) atmosphere at a rate of 10 °C/min between 25 and 600 °C. The structures and crystallinities of the various PLA-based fabrics were investigated using an X-ray diffractometer (XRD, XRD-7000, Shimadzu, Kyoto, Japan). Using XRD equipment, the specimens were studied in the 2–80 2θ-degree range at a continuous scanning rate of 2°/min.

#### 2.5.3. Wettability

To examine the hydrophilicity of the NF textiles, a contact angle (CA) goniometer (OCA 15EC, Data Physics) was utilized. Deionized water droplets (5 ± 0 μL) were placed on the membranes to calculate the droplet angle between the membrane surface and the liquid. An average of ten or more CA measurements were taken at various sites for each sample.

#### 2.5.4. Mechanical Properties

Tensile testing was performed on the fabricated textiles using an Instron apparatus (ElectroPuls, Buckinghamshire, UK). The samples were evaluated at a rate of 5 mm/min. The rectangular test samples were cut according to ASTM D882, with dimensions of 20 × 45 mm. To facilitate handling, each sample was encased in a paper frame. Once the specimens were grasped within the apparatus, the paper frame was sliced using scissors so that the NFs were the only materials remaining between the grips. The stress–strain curve was used to examine the mean ultimate tensile strength.

#### 2.5.5. Antibacterial Properties

Agar diffusion was used to measure the antimicrobial properties of the textiles containing colloidal silver [28,29]. The Gram-positive bacterial strains of *Streptococcus mutans* (S.M), *Staphylococcus aureus* (S.A), *Streptococcus pneumoniae* (S.P), and *Bacillus subtilis* (B.S) were chosen, while the Gram-negative bacterial strain of *Klebsiella pneumoniae* (K.P) was chosen from the list of global priority pathogens (GPP). AgNPs were included in the electrospun NF fabric at a concentration of 20% *v*/*v*, whereas kanamycin was utilized as the conventional antibiotic at the same concentration. The Pharmaceutical Microbiology Department at King Saud University’s College of Pharmacy in Riyadh, Saudi Arabia, developed the microbial cultures. Each microbial strain’s pure colonies were cultivated for 24 h at 37 °C in Mueller–Hilton broth, and 0.5 McFarland standard cultures were made on Mueller–Hilton agar plates. The discs were filled with freshly prepared electrospun samples and standard kanamycin before being placed on an agar plate. The solutions of all electrospun samples were prepared using dimethyl sulfoxide (DMSO). A blank disc (contains DMSO only) was used as a negative control. The zones of inhibition were evaluated following a 24 h incubation period. Each experiment was performed in triplicate.

The antibacterial activity of all samples was also evaluated using the OD600 method. The antibacterial activity of kanamycin (standard) and test samples (M1, M2, M3, and M4) was evaluated against five bacterial strains (B.S, S.P, S.A, S.M, and K.P) using the OD600 absorbance method. Serial dilutions of the samples (2 to 10 µg/mL) were prepared in a 96-well microtiter plate, with each concentration tested in triplicate. Bacterial cultures were adjusted to 0.5 McFarland standard and inoculated into the wells. After 18 h of incubation at 37 °C, OD600 values were measured to assess bacterial growth and % inhibition was calculated using its standard formula reported in the literature [30].

#### 2.5.6. Determination of Minimum Inhibitory Concentration (MIC)

The turbidometric assay was employed to present the inhibitory effects of samples M1, M2, M3, and M4 against each bacterial strain. Standard kanamycin was used as a positive control. Every step of the Clinical and Laboratory Standards Institute (CLSI) broth microdilution protocol was adhered to [28]. A sterile 96 micro-titer plate was utilized for the assay for each bacterial culture. The negative control had no bacteria on it, while the positive control had kanamycin. The test samples M1–M4 were placed into the first well and then diluted twice more until they reached the seventeenth well; the last well was devoid of both germs and drugs (negative control). After that, 5 μL of a diluted bacterial suspension (1.5 × 10^6^ cells/mL) was added to each well (except from the negative control) and thoroughly mixed. Microdilution was carried out in triplicate for every kind of bacterial species. Following an overnight incubation at 37 °C, growth was seen. The lowest concentration prior to turbidity was recorded as the MIC.

## 3. Results and Discussion

### 3.1. Morphological Study

**Figure 2:**
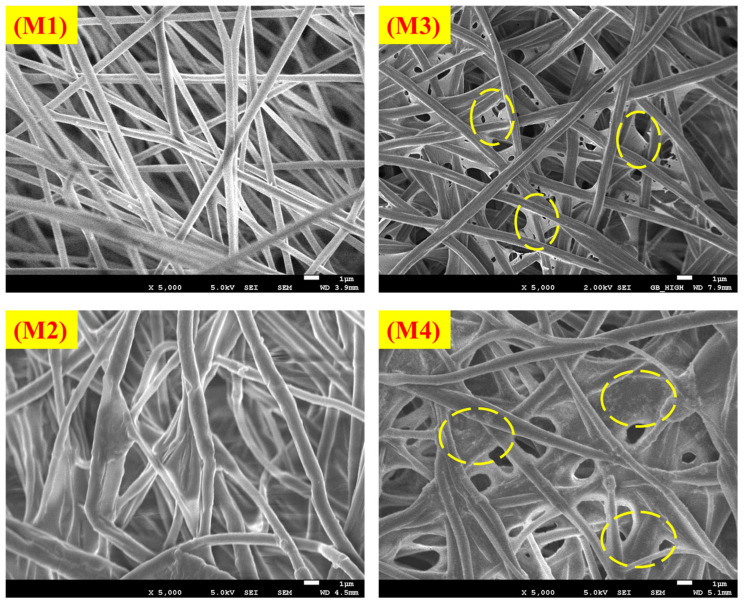
FE-SEM morphological images of the prepared PLA-based nanofibrous electrospun fabrics: (**M1**–**M4**).

**Figure 3:**
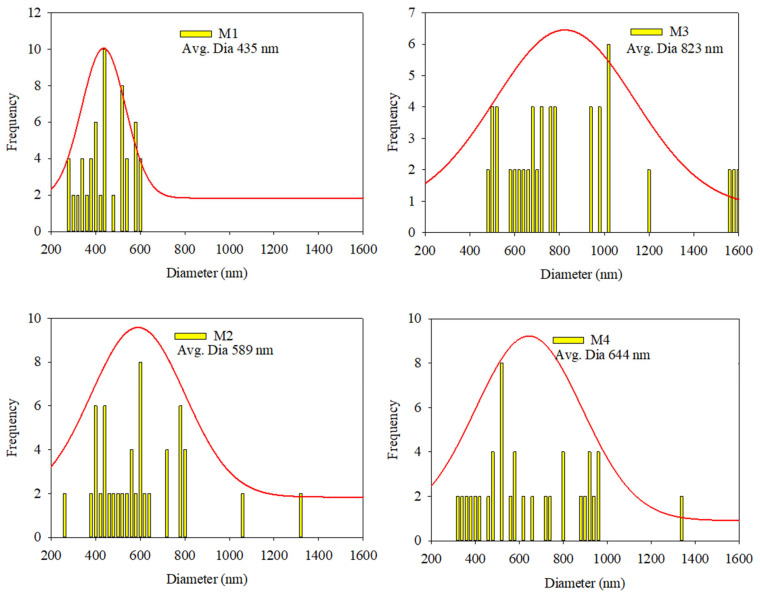
Average diameters and normal distributions of samples M1, M2, M3, and M4.

Morphological analyses were performed to evaluate the fibrous structures of the fabrics. These studies confirmed that the fibrous structure remained intact after the addition of the AgNPs. The fibrous structures at 5000× magnification and the normal distribution of the fiber diameters in the PLA-based nanofibrous textiles are shown in Figure 2 and Figure 3, respectively. The nanostructures of the membranes in samples M1, M2, M3, and M4 displayed uniform, smooth, and beadless fibers, suggesting that the electrospinning parameters were unaffected by the incorporation of AgNPs. The nanofibrous fibers examined in this study were notable for their smoothness and absence of beads or droplets. Using the built-in FE-SEM software (PC-SEM Ver2, 0, 0, 8.), the average diameters for M1, M2, M3, and M4 were determined to be 435, 590, 823, and 644 nm, respectively. Compared to the pure sample shown in Figure S1, the reduction in the mean diameter observed in M1 and M2 was attributed to the presence of AgNPs. This decrease was attributed to the repellent force between the nanoparticles, which decreases the entanglement of the polymer chains. In the M3 and M4 samples, the nanofiber surfaces were free of beads. Nevertheless, the fibers showed a propensity to stick together (highlighted by yellow circles in Figure 2). This adhesion occurred even though identical manufacturing conditions were employed for all samples. As a result, the fibers lacked proper orientation, became thicker, and exhibited an irregular distribution, as shown in Figure 3 [31].

### 3.2. Elemental Analysis

**Figure 4:**
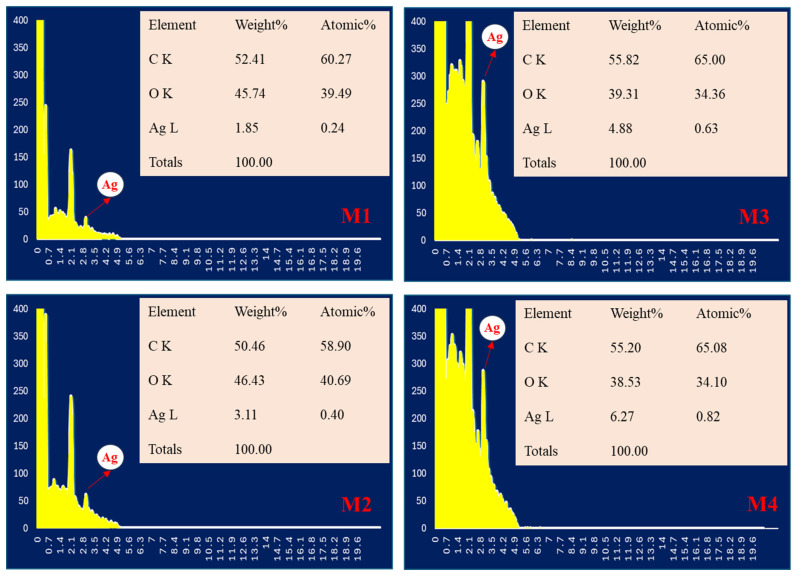
EDX results of the Ag spectrum of the prepared nanofibrous electrospun cloth as evidence of the presence of Ag in the samples.

The elemental compositions of the PLA-based nanofibrous textiles after the incorporation of AgNPs were analyzed using EDX spectrum curves. Figure 4 depicts the EDX elemental spectra and elemental contents of fabric samples M1–M4. The EDX spectra and elemental analysis in Figure 4 confirm the presence of colloidal silver in all samples. When AgNPs were integrated into M1 in the pre-electrospinning stage, the elemental contents were 54.41% carbon (C), 45.74% oxygen (O), and 1.85% Ag. With the integration of the Ag hydrophilic membrane via pre-electrospinning (M2), the Ag content increased, whereas the carbon content decreased. In contrast, in samples M3 and M4, the presence of Ag increased with the incorporation of Ag after electrospinning via solution casting. The highest Ag content and spectrum peak were observed for sample M4, with a slight decrease in the carbon content. This indicates a good interaction between the Ag and the membrane surface. The peak of the platinum coating was not counted and removed, and atomic elemental analysis was performed. Silver colloids were successfully integrated into the membranes; however, the incorporation of AgNPs through solution casting resulted in a higher quantity of Ag compared to the pre-electrospinning stage. The higher Ag content observed in samples M3 and M4 can be attributed to the presence of PEG-PPG-PEG, which enhanced the retention and absorption of the Ag solution within the nanofibrous structure. PEG-PPG-PEG functions by reducing the attractive forces among NPs, increasing the steric distance between them and improving the hydrophilicity. Consequently, this led to an improved dispersion of the AgNPs and a higher concentration of Ag detected in the elemental analysis [32].

### 3.3. Wettability

**Figure 5:**
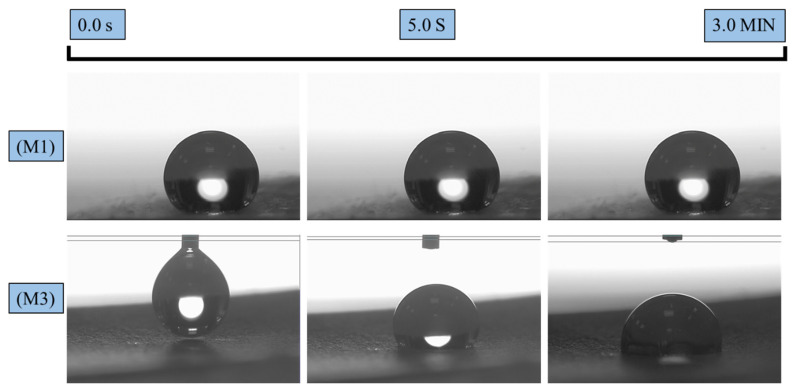
Contact angles of PLA-based textiles: (**M1**) PLA-Ag-E and (**M3**) PLA-Ag-S, initially and after 300 s.

**Figure 6:**
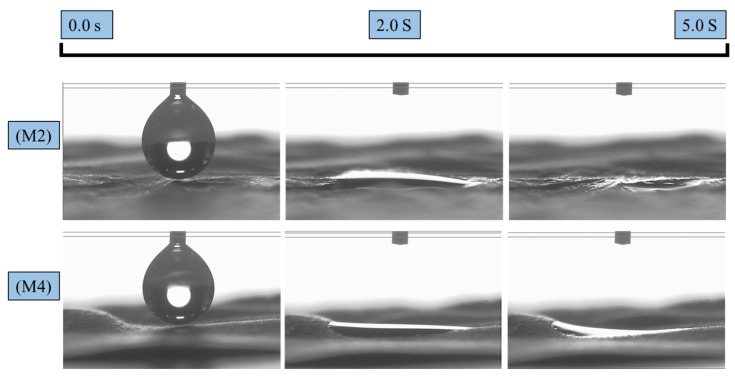
Contact angles of PLA-based textiles: (**M2**) PLA-PEG-AG-E and (**M4**) PLA-PEG-AG-S, initially and after 5 s.

Figure 5 and Figure 6 illustrate the effects of incorporating AgNPs into the electrospun textiles. Contact angle measurements were conducted for all hydrophobic and hydrophilic textiles. The wettability behaviors of the pure PLA fabric with AgNPs added during the pre-and post-electrospinning stages (M1 and M3) are shown in Figure 5. M1 exhibited a stable water droplet with a CA of 128° after 3 min, demonstrating PLA’s hydrophobic nature. By contrast, M3 exhibited a reduced CA of 92° for the same duration. This decrease in hydrophobicity may be attributed to the direct interaction between the silver colloidal solution and the PLA membrane surface. During the solution casting of colloidal silver, the solution contained low-surface-energy liquids, such as isopropanol, which infiltrated the pure PLA surface and diminished its wettability. Figure 6, however, demonstrates the hydrophilic characteristics of samples M2 and M4, where the surface CA of the water droplet rapidly reached approximately 0°, as the membrane surface completely absorbed the water within 2 s. This phenomenon occurred because the addition of PEG and AgNPs to PLA created a hydrophilic environment that rapidly attracted water molecules [33]. This enhanced hydrophilicity contributes to protecting textiles against bacterial activity, as water can penetrate the fiber matrix and interact with antibacterial agents, such as silver.

### 3.4. X-ray Diffraction

**Figure 7:**
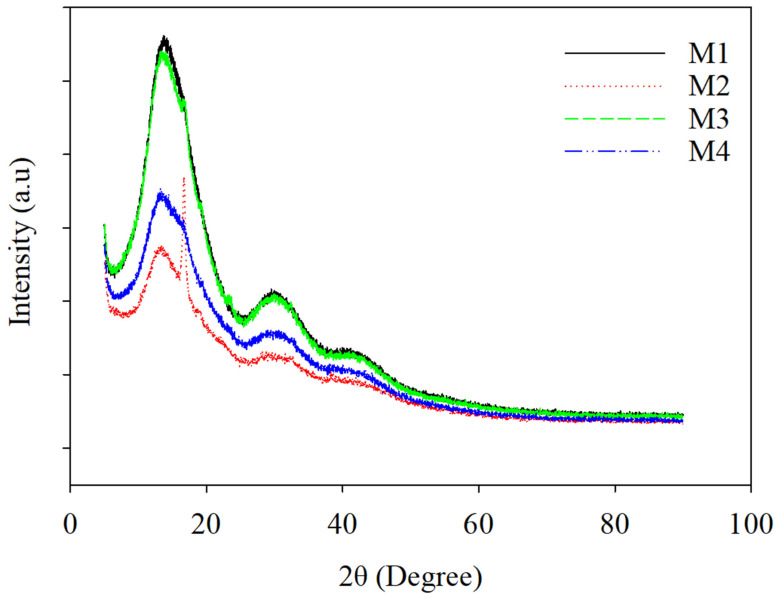
XRD results of the prepared nanofibrous electrospun cloth: (M1) PLA-Ag-E, (M2) PLA-Ag-S, (M3) PLA-PEG-Ag-E, and (M4) PLA-PEG-Ag-S.

Figure 7 depicts the X-ray diffraction (XRD) patterns of the PLA-based electrospun nanofiber textiles, which were categorized based on their hydrophilicity and the method of AgNP incorporation (M1, M2, M3, and M4). The XRD curves for these multi-functional electrospun textiles exhibit prominent diffraction peaks between 2*θ* = 13 and 19°, confirming the amorphous structure of PLA and PEG-PPG-PEG in the membranes, corresponding to the (203) crystal plane [34]. The integration of PEG-PPG-PEG and AgNPs resulted in decreased diffraction peak intensity, indicating reduced crystallization in the PLA/PEG-PPG-PEG/AgNP samples (M3 and M4). Moreover, the peak broadening observed in M3 and M4 suggests an increase in the amorphous regions due to the PEG-PPG-PEG content in the nanofiber fabrics. Compared to the pristine sample, the XRD peaks for the PLA blends were nearly identical to those of the pure hydrophobic PLA and hydrophilic PLA/PEG-PPG-PEG membranes, as shown in Figure S2. However, the diffractive intensities of the blends increased due to the presence of AgNPs. Minor diffraction peaks were detected in all samples, which could be attributed to the microcrystallinity of the PLA, PEG-PPG-PEG, and AgNPs. Notably, a distinct and broad peak at around 38–44° was observed in the composites containing AgNPs, which can be ascribed to the 111 and 200 crystallographic planes of the face-centered cubic (FCC) silver crystals, respectively [34]. The increased crystallinity in samples M3 and M4 was evident from the decreased amorphous region area around 2*θ* = 13 and 19°. This enhancement resulted in significant reductions in the amorphous region area, with M3 showing a 62% decrease and M4 demonstrating a 49% decrease when compared to samples M1 and M2, respectively.

### 3.5. Thermal Analysis

**Figure 8:**
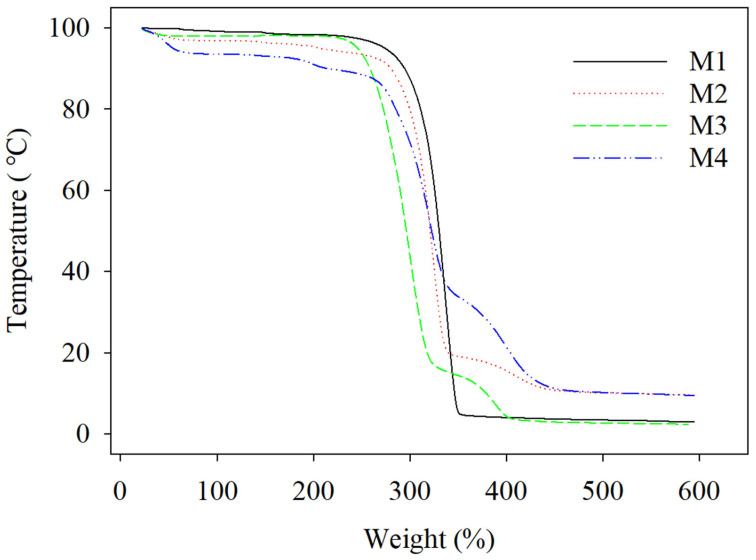
TGA results of the prepared nanofibrous electrospun cloth: (M1) PLA-Ag-E, (M2) PLA-Ag-S, (M3) PLA-PEG-Ag-E, and (M4) PLA-PEG-Ag-S.

Figure 8 illustrates the thermal analysis results for samples M1, M2, M3, and M4 as TGA curves. The initial examination of these curves focused on M1 and M3, which were pure PLA-based samples containing AgNPs. M1 began to decompose, presenting an onset temperature of 312 °C and reaching its peak breakdown rate at 337 °C. In contrast, M3 began to degrade at an onset temperature of 303 °C, with its maximum thermal breakdown rate occurring at approximately 324 °C. The data suggest that the addition of the AgNPs via solution casting not only reduced the wettability properties but also reduced the thermal stability, which could be due to a correlation between the crystallinity of the PLA materials and their thermal stability, with lower crystallinity corresponding to lower thermal stability.

In contrast, the presence of PEG-PPG-PEG in the M2 and M4 samples reduced the onset temperature of the PLA phase to 272 °C for M2 and to 290 °C for M4. These samples exhibited two decomposition peaks. The first peaks occurred at approximately 304 °C for M2 and at 320 °C for M4, signifying the thermal degradation of the PLA matrix. The second peaks indicate the thermal breakdown of PEG-PPG-PEG at 393 and that of M2 and M4 at 411 [35]. This fluctuation in the PLA peak may be attributed to the lubrication of the PLA molecules due to the PEG-PPG-PEG content. Therefore, M2 and M4 demonstrated lower thermal decomposition compared to M1 and M3, possibly due to the PEG-PPG-PEG plasticizing the PLA and reducing its crystallinity [11]. Overall, the addition of AgNPs decreased the thermal stability of the PLA-based textile compared to that of the pristine sample, as shown in Figure S3.

### 3.6. Mechanical Strength

**Figure 9:**
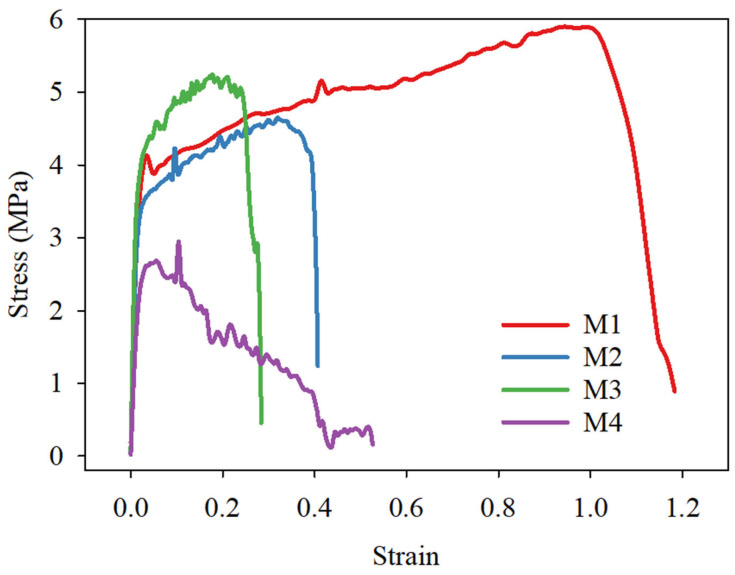
Tensile test results of the prepared nanofibrous electrospun fabric for samples M1, M2, M3, and M4.

The mechanical properties of the PLA-based electrospun textiles were evaluated through tensile strength analysis, as illustrated in Figure 9. The stress–strain curves revealed three distinct phases in the mechanical behavior of the nanofiber garments. Initially, a linear elastic region with a steep slope was observed, followed by a gradual increase in stress levels. The final phase was characterized by rapid failure, resulting in a significant reduction in stress. Samples M1 and M3 exhibited comparable ultimate tensile strengths of 5.9 and 5.6 MPa, respectively, due to their similar material compositions. However, their elongation percentages differed significantly, with M1 reaching 120% and M3 only 40%. This disparity can be attributed to the post-electrospinning incorporation of AgNPs in M3 through solution casting, which introduced brittleness to the material. Samples M2 and M4, containing hydrophilic PEG-PPG-PEG components, demonstrated lower tensile strengths of 4.8 and 2.3 MPa, respectively. Their elongation percentages were 30% for M2 and 39% for M4. The post-electrospinning treatment applied to M4 resulted in reduced tensile strength compared to M2. The observed decrease in the mechanical properties of the post-treated samples (M3 and M4) correlates with their diminished thermal characteristics. This correlation may be explained by the lower crystallinity of these samples, as evidenced by the XRD analysis. The post-electrospinning treatment likely disrupted the molecular alignment and crystalline structure of the fibers, leading to reduced mechanical and thermal performance. These findings emphasize the need to balance the incorporation of AgNP functional additives with preserving mechanical properties in electrospun textiles. Future research should aim to optimize post-electrospinning treatments to reduce adverse effects on the mechanical and thermal properties while maintaining functionality.

### 3.7. Antibacterial Properties

The in vitro antimicrobial activity of the electrospun NF textiles was qualitatively evaluated based on the presence or absence of the inhibitory zones. The positive antibacterial action in the M2, M3, and M4 samples was observed to be effective against S.M, B.S, S.A, and S.P (Table 2).

**Figure 10:**
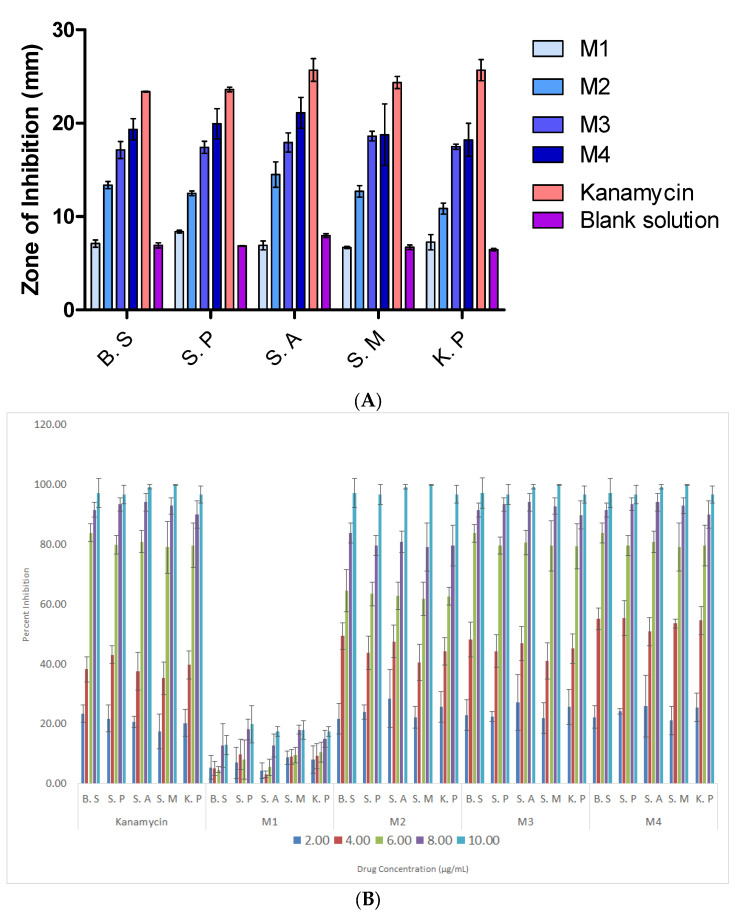

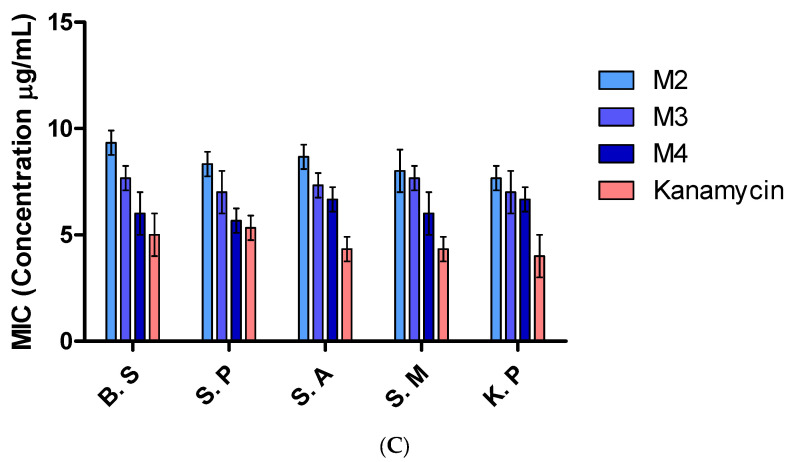
(**A**) Antibacterial activity of the prepared electrospun nanofibrous fabric samples (zone of inhibition), (**B**) % inhibition by OD600 method, and (**C**) MIC values of samples M1–M4 and standard kanamycin.

All microorganisms were highly susceptible to the antibacterial activity of sample M4, such as the B.S, S.P, S.A, S.M, and K.P strains, and were identified based on the outcomes of the agar diffusion technique. The inhibition zones for B.S, S.P, S.A, S.M, and K.P were recorded to be 19.30, 20.11, 20.09, 19.09, and 16.86 mm, respectively, as shown in Figure 10A. This discovery is most likely the result of the hydrophilicity and surface area of the NFs, which enabled Ag to interact with the bacteria more easily. The antibacterial action of silver nanoparticles in the hydrophilic samples could be mediated by reactive oxygen species that damage bacterial membranes, allowing internal components to seep out and causing damage to proteins and DNA, eventually resulting in the death of the bacterium. In the presence of the pure PLA hydrophobic M1 sample (Figure 10A), no significant activity was seen for any bacteria. The inhibitory effects of M1 were almost similar to the negative control, showing no antibacterial activity. Some bacteriostatic effects of blank disc were associated to the DMSO, as DMSO has also been reported to have some antimicrobial properties [35].

The zone of inhibition results was also confirmed using an OD600 method. The results of % inhibition against different concentrations of M1–M4 and standard kanamycin are shown in Figure 10B. The results demonstrate the concentration-dependent antibacterial activity of the tested samples. Kanamycin exhibited the highest percent inhibition (~100%) at 10 µg/mL across all bacterial strains, validating its strong antibacterial potency. Among the test samples, M4 showed the most promising activity, closely mirroring kanamycin’s performance, particularly at concentrations of 8 and 10 µg/mL, where it achieved >90% inhibition against B.S, S.P, and S.A. M3 also displayed significant activity, though slightly less effective than M4. Conversely, M2 demonstrated moderate activity with partial inhibition across the bacterial strains, while M1 was the least effective. The maximum inhibitory effects were observed in M4 compared to other samples. The results of the OD600 method were in accordance with the results of zone of inhibition. These findings highlight the broad-spectrum potential of the tested compounds against both Gram-positive bacteria (B.S, S.P, S.A, and S.M) and Gram-negative bacteria (K.P), making them promising candidates for further antimicrobial applications.

The results of MIC data for M1–M4 and standard kanamycin are presented in Figure 10C. Based on the provided MIC data, the tested samples M2, M3, and M4 demonstrate varying levels of antimicrobial activity when compared to the standard, kanamycin. M2 showed the highest MIC values across all microorganisms, with averages ranging from 7.67 ± 0.58 to 9.33 ± 0.58 µg/mL, indicating lower potency. M3 displayed slightly better activity, with MIC values ranging from 7.00 ± 1.00 to 7.67 ± 0.58 µg/mL, showing moderate effectiveness. M4, on the other hand, exhibited MIC values between 5.67 ± 0.58 and 6.67 ± 0.58 µg/mL, closely approximating the activity of kanamycin, which had MIC values ranging from 4.33 ± 0.58 to 5.33 ± 0.58 µg/mL. Compared to standard kanamycin, the inferior antibacterial effects of tested materials would be possible due to the sustained-type release of Ag ions from the tested materials. Among the tested samples, M4 demonstrated the most comparable efficacy to kanamycin, particularly against S.P and S.M, where its MIC values were almost equivalent to those of the standard. These results highlight M4 as a promising candidate for further investigation, while M2 and M3 showed lower antimicrobial potential.

**Figure 11:**
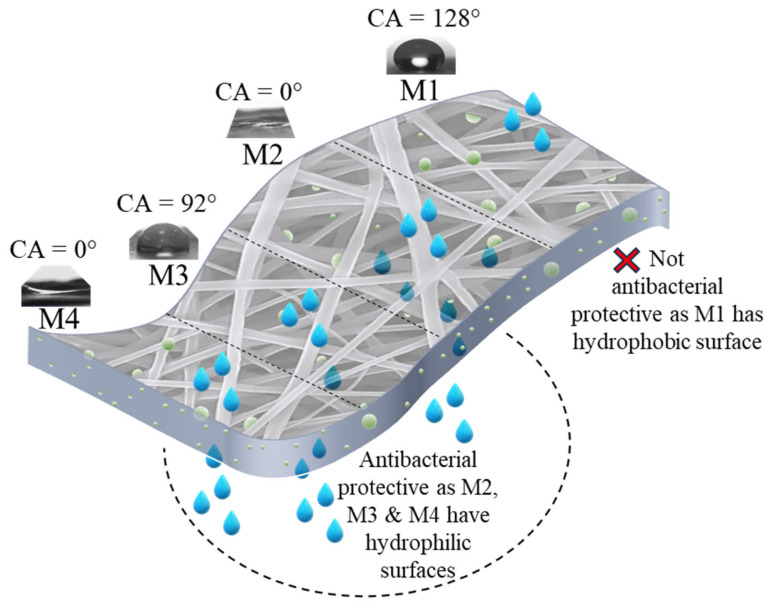
Schematic illustration of how hydrophilic surfaces absorb water and interact with AgNPs to enhance antibacterial properties.

The presence of silver colloids in hydrophilic membranes is crucial for combatting bacterial agents; however, the integration of AgNPs into these membranes also plays a significant role, as shown in Figure 11. Samples M3 and M4, which were prepared using post-electrospinning to integrate AgNPs, demonstrated the most effective antibacterial properties. Interestingly, even the pure PLA exhibited strong antibacterial characteristics owing to the solution casting process, which enhanced the surface hydrophilicity and improved the integration of AgNPs into the PLA matrix. Sample M4, featuring hydrophilic PLA with post-electrospinning modification, outperformed sample M2, in which AgNPs were incorporated through pre-electrospinning. These findings suggest that the post-electrospinning method of AgNP incorporation is advantageous for enhancing the antibacterial properties of the nanofibrous membranes.

## 4. Conclusions

This research successfully integrated microwave-assisted colloidal silver into PLA-based nanofibrous textiles using pre- and post-electrospinning techniques to improve the antibacterial properties, with the objective of creating advanced materials for biomedical and protective apparel applications. The primary results included the generation of consistent nanofibers with diameters ranging from 435 to 823 nm, the verification of AgNP incorporation through EDX analysis, improved nanofiber hydrophilicity with the addition of a PEG-PPG-PEG copolymer, decreased crystallinity and lower decomposition temperatures in the hydrophilic samples, similar tensile strengths across the samples with decreased elongation in the post-treated samples, and improved antibacterial effectiveness against various bacterial strains in the AgNP-incorporated nanofibers. Interestingly, even the pristine PLA nanofibers integrated with AgNPs subjected to post-electrospinning treatment displayed antibacterial properties due to the induction of hydrophilicity. This investigation offers valuable insights into the development of AgNP-incorporated PLA-based nanofibrous textiles and their potential applications. These results establish a foundation for additional research and development in this area, facilitating the development of advanced materials with enhanced antibacterial properties for various biomedical and protective applications.

## Figures and Tables

**Table 1:** Sample coding and methods for integrating AgNPs into PLA-based nanofibrous hydrophobic and hydrophilic textiles.

Sample Names	PLA (wt.%)	PEG-PPG-PEG (wt.%)	Silver Colloidal	Method of AgNP Integration	Electrospinning Dope Solution
M1	100	0	20 (*v*/*v*. %)	Electrospinning (E)	PLA/AgNPs
M2	80	20	20 (*v*/*v*. %)	Electrospinning (E)	PLA/PEG-PPG-PEG/AgNPs
M3	100	0	0.083 mL/cm^2^	Solution casting (S)	PLA
M4	80	20	0.083 mL/cm^2^	Solution casting (S)	PLA/PEG-PPG-PEG

**Table 2:** Antibacterial activity (inhibition zone) values of hydrophilic and hydrophobic electrospun NF fabrics against Gram-positive and Gram-negative bacterial strains.

Samples	Inhibition Zone (mm) ± SD, n = 3				
	B.S	S.P	S.A	S.M	K.P
M1	6.63 ± 0.80	6.57 ± 1.02	6.83 ± 1.16	6.69 ± 1.43	6.75 ± 0.84
M2	13.25 ± 0.85	12.26 ± 0.44	13.51 ± 1.74	12.49 ± 0.91	10.36 ± 1.55
M3	16.91 ± 2.02	16.83 ± 0.70	18.54 ± 1.77	17.99 ± 1.10	19.22 ± 1.51
M4	19.30 ± 2.63	20.11 ± 2.02	20.09 ± 2.10	19.09 ± 2.95	16.86 ± 2.04
Kanamycin	22.80 ± 0.93	24.04 ± 1.47	25.42 ± 1.33	24.40 ± 0.93	25.81 ± 1.23
Blank	7.06 ± 0.46	6.96 ± 0.27	7.61 ± 0.35	6.88 ± 0.42	6.60 ± 0.30

## Data Availability

The original contributions presented in this study are included in the article/Supplementary Materials; further inquiries can be directed to the corresponding authors.

## Supplementary Materials

The following supporting information can be downloaded at: https://www.mdpi.com/article/10.3390/polym16243613/s1, Figure S1: FE-SEM images of (a) pristine PLA and (b) PLA/PEG-PPG-PEG NFs at 5000x magnification, and normal fiber diameter distribution with the average fiber diameter of (b) pristine PLA and (c) PLA/PEG-PPG-PEG NFs; Figure S2: XRD results of the pristine PLA and PLA/PEG-PPG-PEG NFs to compared with sample M1, M2, M3 and M4; Figure S3: TGA results of the prepared pristine nanofibrous electrospun clothes.
