# Supplementary material for: The Integration of Microwave-Synthesized Silver Colloidal Nanoparticles into Poly (Lactic Acid)-Based Textiles as Antimicrobial Agents via Pre- and Post-Electrospinning Processes
Source: Polymers (Basel). 2024 Dec 23;16(24):3613. doi: 10.3390/polym16243613 (PMC11678830; doi:10.3390/polym16243613)
Supplement: Supplementary file 1 [file polymers-16-03613-s001.zip › polymers-3309503-supplementary.pdf]

Supplementary Information

Integration of Microwave-Synthesized Silver Colloidal Nanoparticles into Poly (Lactic Acid)-Based Textiles Via Pre- and Post-Electrospinning Processes as Antimicrobial Mediator

Muhammad Omer Aijaz[1,2,#], Ibrahim A. Alnaser[1,2,3,*,#], Md. Irfanul Haque Siddiqui[2,3], Mohammad Rezaul Karim[1,2,*,#]

[1]Center of Excellence for Research in Engineering Materials (CEREM), Deanship of Scientific Research (DSR), College of Engineering, King Saud University, Riyadh 11421, Saudi Arabia; maijaz@ksu.edu.sa (M.O.A.)

[2]The King Salman Center for Disability Research, Riyadh 12512, Saudi Arabia

[3]Department of Mechanical Engineering, College of Engineering, King Saud University, Riyadh 11421, Kingdom of Saudi Arabia; msiddiqui2.c@ksu.edu.sa (M.I.S.)

*Correspondence: ianaser@ksu.edu.sa (I.A.A.); mkarim@ksu.edu.sa (M.R.K.)

[#]Authors have made equal contributions.

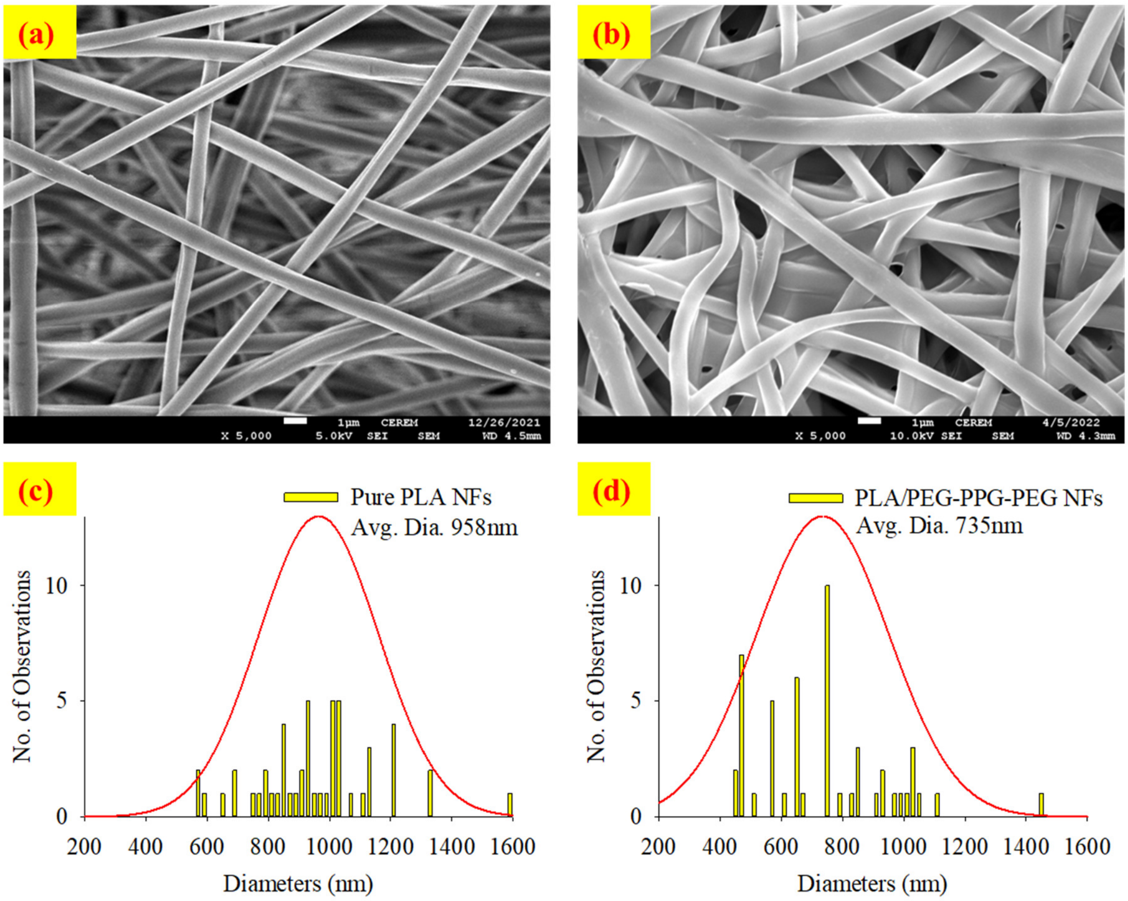

Figure S1. FE-SEM images of (a) pristine PLA and (b) PLA/PEG-PPG-PEG NFs at 5000x magnification, and normal fiber diameter distribution with the average fiber diameter of (b) pristine PLA and (c)PLA/PEG-PPG-PEG NFs.

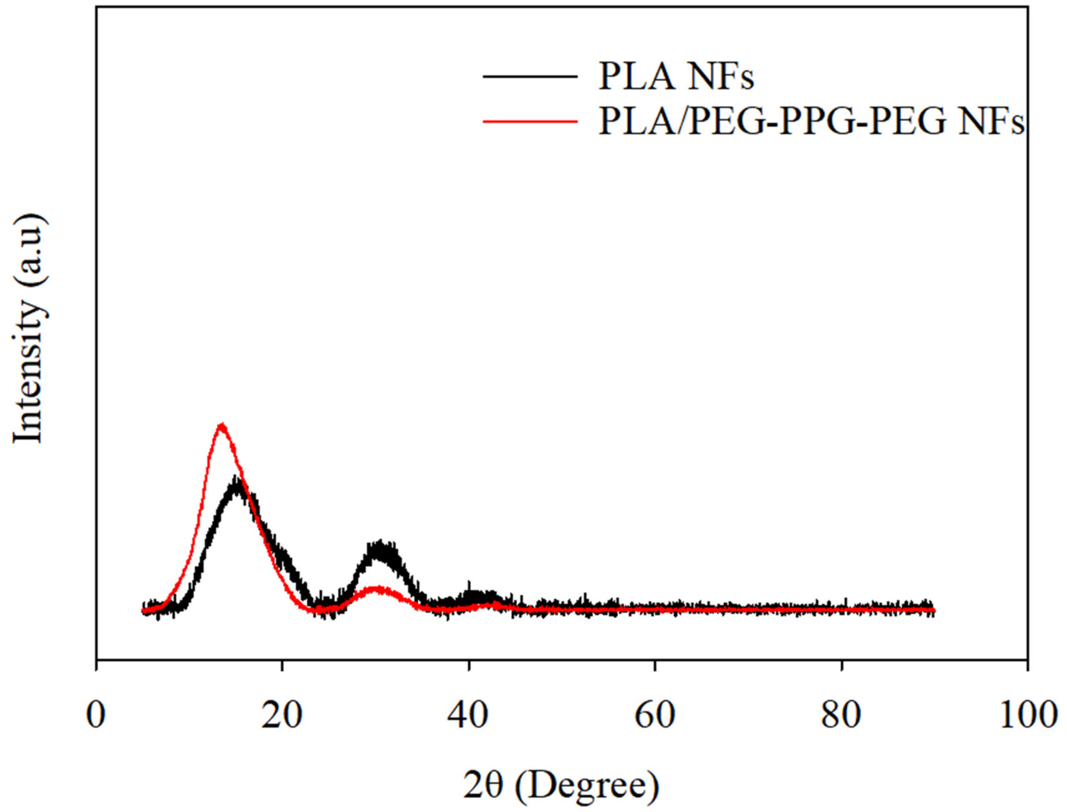

Figure S2. XRD results of the pristine PLA and PLA/PEG-PPG-PEG NFs to compared with sample M1, M2, M3 and M4.

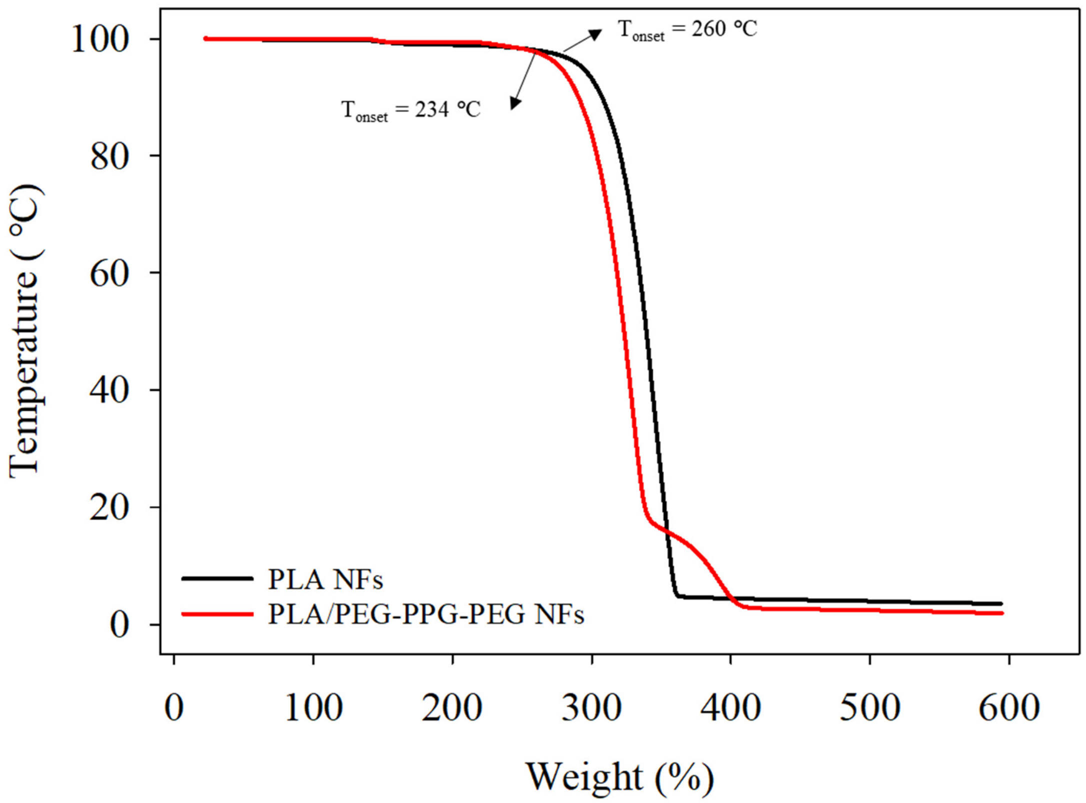

Figure S3. TGA results of the prepared pristine nanofibrous electrospun clothes
